# GraphML specializations to codify ancestral recombinant graphs

**DOI:** 10.3389/fgene.2013.00146

**Published:** 2013-08-07

**Authors:** James R. McGill, Elizabeth A. Walkup, Mary K. Kuhner

**Affiliations:** Department of Genome Sciences, University of WashingtonSeattle, WA, USA

**Keywords:** graphML, ARG, ancestral recombination graph, Newick, XML

## Abstract

Software which simulates, infers, or analyzes ancestral recombination graphs (ARGs) faces the problem of communicating them. Existing formats omit information either about the location of recombinations along the chromosome or the position of recombinations relative to the branching topology. We present a specialization of GraphML, an XML-based standard for mathematical graphs, for communication of ARGs. The GraphML <node> type is specialized to contain the node type, time, recombination location, and name. The GraphML <edge> type is specialized to contain the ancestral material passed along that edge. This approach, which we call ArgML, retains all information in the original ARG. Due to its use of established formats ArgML can be parsed, checked and displayed by existing software.

## Introduction

Phylogenetic trees used to represent the histories of species or populations are usually communicated using the Newick format described in Olsen (1990). Ancestral recombinant graphs (ARGs) (Griffiths and Marjoram, 1997) are directed acyclic graphs which generalize phylogenetic trees to allow recombination. Griffiths' specification of the ARG gives both the time and branching structure associated with each recombination event and the ancestral material inherited along each branch. However, this has not been easy to accommodate within current formats for communicating phylogenies.

Two approaches have been used. *Interval-tree* representations break the chromosome into non-recombining segments, specifying the Newick tree of each segment along with the segment boundaries. This approach is used by the *ms* program of Hudson (2002) and other data simulators as it provides sufficient information for simulation of data on an ARG, but it loses information about the number, time, and topological location of recombination events. *Directed-graph* representations [used by Extended Newick (Cardona et al., 2008) among others (see for a review, Arenas et al., 2010)], store the ARG as a directed graph with no specification of which material is inherited along each edge. This is useful in analysis of hybridization, but it loses information about which parts of the chromosome were inherited from each ancestor. While the NeXML standard (Vos et al., 2012) discusses the potential use of NeXML for ARGs, it does not specify the tags needed to add ancestral information, so currently offers only the directed-graph representation. In this paper we propose a format based on the directed-graph approach but specifying the ancestral material inherited along each edge. All details of the ARG can be reconstructed from this format.

The GraphML standard (Bandes et al., 2001) was developed to codify graph structures in terms of nodes and edges. Tools such as Mathematica (Wolfram, 2003) and Gephi (Bastian et al., 2009) provide methods for reading and plotting GraphML files, though they display only connectivity as they have no concept of time ordering. Since GraphML is based on XML (Bray et al., 2008), GraphML files can be parsed and error-checked by XML-handling software. Thus, programs wishing to read or write GraphML can make use of existing XML libraries such as TinyXML (Thomason, 2013).

**Figure A1:**
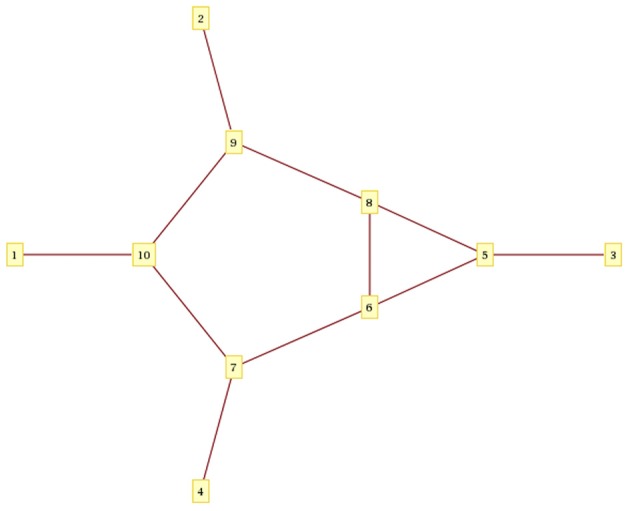
**Mathematica version of Figure 1 recombinant graph**.

Motivated by the need of our program LAMARC (Kuhner, 2006) to store and communicate ARGs, we have developed ArgML, a specialization of GraphML which adds time and ancestral material information. We propose it as a standard format for communicating ARGs between programs. ArgML files can be read directly by Mathematica (an example is shown in Figure A1) and will be read and written by an upcoming version of Lamarc.

## Methods

To express coalescent times, node types, and sites transmitted, we leveraged GraphML's general-purpose node and edge annotation capability as follows. To the <node> tag we added four fields:<node_type>, the kind of node (Tip, Rec, Coal); <node_label>, the (optional) name of the node; <node_time>, the time of the node (relative to the time at the tips); and <rec_location>, the chromosomal location of the recombination represented by this node, if any. To the <edge> tag we added <live_sites>, giving the ancestral material transmitted along that <edge>. The contents of <live_sites> are one or more entries of the form [firstsite:lastsite+1). This [x:y) notation is a standard convention for half-open intervals (e.g., Austern, 1999) and indicates that the first site of the recombinational interval is x and the last site is one site before y; site y itself is not included. If the ancestral material contains more than one discontinuous segment, this is written as <live_sites> [w:x) [y:z) </live-sites>.

These new keys are defined within the GraphML source file (see Appendix) and can be handled by an XML parser such as TinyXML (Thomason, 2013) without further intervention.

Time information could be expressed either as a branch length (as in Newick format) or a node time. We have found that branch length representation of a strict clocklike tree is prone to numerical precision issues leading to violations of the clock when branch lengths are summed. Use of node times avoids this problem.

## Limitations

We assume that an ARG is time-ordered and clocklike. Non-clocklike trees are difficult to use in the ARG context as time information is needed to distinguish the lineages contributing to a recombination from the resulting recombinant lineage. Therefore, violation of the molecular clock in an ARG is best represented by a multiplier on the time-based branch length, not by a mutation-based non-clocklike branch length. Such a multiplier could readily be added to ArgML.

Users of ArgML should be aware that the clock requirement cannot be checked by GraphML parsers and should be checked by special-purpose code in programs reading or writing ARGs.

We also assume that the ARG is fully specified with the locations of all recombinations. Graphs without locational information can arise from hybridization where the contribution of each parental species to the hybrid is not known. They could be straightforwardly coded in GraphML but will not be substitutable for ARGs in most applications (for example, whereas an ARG can be decomposed into interval trees, this is not true for a hybridization graph).

The ArgML format does not represent gene conversion or multiple crossovers in the same meiosis. These events could be coded as two or more recombinations occurring at the same time, although this would impose a fictitious ordering among what are actually components of the same event.

Currently no tool exists to display time ordered ArgML trees. It is to be hoped that someone will create such a tool in the future.

## Example

**Figure 1:**
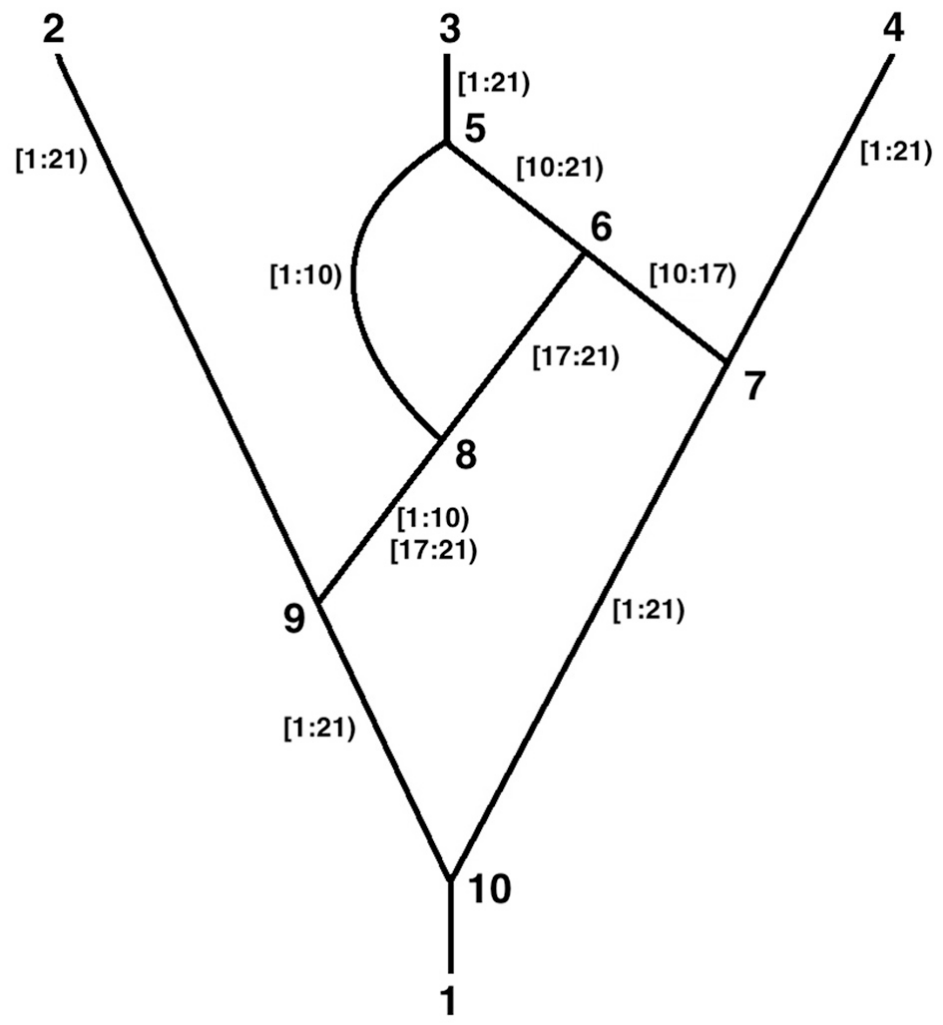
**A recombinant graph**.

Consider the following time ordered ARG (Figure 1). The tips are labeled 2, 3, and 4, the root is 1, the coalescences are 7, 8, 9, and 10, and the recombinations are 5 and 6. Ancestral material transmitted along each edge is indicated. There are 20 sites in the ancestral material. Recombinations occur at the link between two sites and there cannot be links before the first site or after the last, therefore there are 19 links.

Thus, recombination 6 above is defined by:


<node id=“6”>
  <data key=“node_type”>Rec</data>
  <data key=“node_time”>0.2</data>
  <data key=“rec_location”>17</data>
</node>
<edge source=“7” target=“6”>
  <data key=“live_sites”>[10:17)</data>
</edge>
<edge source=“8” target=“6”>
  <data key=“live_sites”>[17:21)</data>
</edge>


The ancestral material transmitted between nodes 8 and 6 above is expressed as [17:21) which is a half open interval and is read as “the segment that begins at site 17 and ends before site 21.” Thus, it contains sites 17, 18, 19, and 20 and the links between them. Similarly [10:17) contains sites 10–16 and their connecting links. To maintain consistency with this half open interval notation, the <rec_location> of the recombination that is between 16 and 17 is numbered 17 and can be thought of as being “before” site 17.

Note in the figure that two discontinuous segments are transmitted between nodes 8 and 9. This is expressed by:


<edge source=“9” target=“8”>
  <data key=“live_sites”>
   [1:10)[17:21)</data>
</edge>


## Conclusions

ArgML augments the well-established GraphML format with all of the information needed to transmit ARGs. A full ARG identical to the original can be drawn from the ArgML representation even if multiple recombinations occurred at the same inter-site link. This specialization allows users to leverage the numerous existing tools that already understand GraphML. Further information needed for handling of ARGs could be readily added to the standard.

### Conflict of interest statement

The authors declare that the research was conducted in the absence of any commercial or financial relationships that could be construed as a potential conflict of interest.

## Appendix

### Appendix 1

Full GraphML Source File for the Ancestral Recombination Graph:

<?xml version=“1.0” ?>

<graphml>
<key id=“live_sites” for=“edge” attr.name=“lvlinks” attr.type=“string” />
<key id=“rec_location” for=“node” attr.name=“rloc” attr.type=“long” />
<key id=“node_type” for=“node” attr.name=“ntype” attr.type=“string” />
<key id=“node_time” for=“node” attr.name=“ntime” attr.type=“double” />
<key id=“node_label” for=“node” attr.name=“nlabel” attr.type=“string” />
<graph id=“myGraph” edgedefault=“undirected”>
<node id=“2”>
<data key=“node_type”>Tip</data>
<data key=“node_time”>0</data>
<data key=“node_label”>tip1</data>
</node>
<edge source=“9” target=“2”>
<data key=“live_sites”>[1:21)</data>
</edge>
<node id=“3”>
<data key=“node_type”>Tip</data>
<data key=“node_time”>0</data>
<data key=“node_label”>tip2</data>
</node>
<edge source=“5” target=“3”>
<data key=“live_sites”>[1:21)</data>
</edge>
<node id=“4”>
<data key=“node_type”>Tip</data>
<data key=“node_time”>0</data>
<data key=“node_label”>tip3</data>
</node>
<edge source=“7” target=“4”>
<data key=“live_sites”>[1:21)</data>
</edge>
<node id=“5”>
<data key=“node_type”>Rec</data>
<data key=“node_time”>0.1</data>
<data key=“rec_location”>10</data>
</node>
<edge source=“6” target=“5”>
<data key=“live_sites”>[10:21)</data>
</edge>
<edge source=“8” target=“5”>
<data key=“live_sites”>[1:10)</data>
</edge>
<node id=“6”>
<data key=“node_type”>Rec</data>
<data key=“node_time”>0.2</data>
<data key=“rec_location”>17</data>
</node>
<edge source=“7” target=“6”>
<data key=“live_sites”>[10:17)</data>
</edge>
<edge source=“8” target=“6”>
<data key=“live_sites”>[17:21)</data>
</edge>
<node id=“7”>
<data key=“node_type”>Coal</data>
<data key=“node_time”>0.3</data>
</node>
<edge source=“10” target=“7”>
<data key=“live_sites”>[1:21)</data>
</edge>
<node id=“8”>
<data key=“node_type”>Coal</data>
<data key=“node_time”>0.4</data>
</node>
<edge source=“9” target=“8”>
<data key=“live_sites”>[1:10)[17:21)</data>
</edge>
<node id=“9”>
<data key=“node_type”>Coal</data>
<data key=“node_time”>0.5</data>
</node>
<edge source=“10” target=“9”>
<data key=“live_sites”>[1:21)</data>
</edge>
<node id=“10”>
<data key=“node_type”>Coal</data>
<data key=“node_time”>0.6</data>
</node>
<edge source=“1” target=“10”>
<data key=“live_sites”>[1:21)</data>
</edge>
</graph>
</graphml>

### Appendix 2

Mathematica Plot of the Ancestral Recombination Graph:

To generate the Mathematica plot below, store the above data as “recombtree.graphml”

Command to read recombtree.graphml and view it:

recplot = Import[“recombtree.graphml”, “Graphics”, VertexLabeling -> True, ImageSize -> {700, 700}]

Note this is purely the connectivity of the tree as Mathematica has no concept of time ordering.
